# Pain Control by Targeting Oxidized Phospholipids: Functions, Mechanisms, Perspectives

**DOI:** 10.3389/fendo.2020.613868

**Published:** 2021-01-25

**Authors:** Beatrice Oehler, Alexander Brack, Robert Blum, Heike L. Rittner

**Affiliations:** ^1^ Wolfson Center of Age-Related Diseases, IoPPN, Health and Life Science, King’s College London, London, United Kingdom; ^2^ Department of Anesthesiology, University Hospital of Heidelberg, Heidelberg, Germany; ^3^ Department of Anesthesiology, University Hospital of Würzburg, Würzburg, Germany; ^4^ Institute of Clinical Neurobiology, Department of Neurology, University Hospital of Würzburg, Würzburg, Germany

**Keywords:** oxidized phospholipids, TRP channel, ion channel, analgesia, pain therapy, nociception, therapeutic antibody, mimetic peptide

## Abstract

Within the lipidome oxidized phospholipids (OxPL) form a class of chemically highly reactive metabolites. OxPL are acutely produced in inflamed tissue and act as endogenous, proalgesic (pain-inducing) metabolites. They excite sensory, nociceptive neurons by activating transient receptor potential ion channels, specifically TRPA1 and TRPV1. Under inflammatory conditions, OxPL-mediated receptor potentials even potentiate the action potential firing rate of nociceptors. Targeting OxPL with D-4F, an apolipoprotein A-I mimetic peptide or antibodies like E06, specifically binding oxidized headgroups of phospholipids, can be used to control acute, inflammatory pain syndromes, at least in rodents. With a focus on proalgesic specificities of OxPL, this article discusses, how targeting defined substances of the epilipidome can contribute to mechanism-based therapies against primary and secondary chronic inflammatory or possibly also neuropathic pain.

## Introduction

Pain is the result of molecular and psycho-behavioural elements triggered by tissue damage (1). In developed countries, the estimated prevalence of chronic pain is about 20% (2, 3). In the new, eleventh international classification of diseases (ICD-11), primary chronic pain has been classified as an independent disease, like low back pain or fibromyalgia, and separated from syndromes where pain is secondary to another underlying disease, like in osteoarthritis or rheumatoid arthritis (4). To improve the quality of life, an effective pain treatment has major impact. Reports by the National Institutes of Health indicate that opioids have been the most prescribed drugs for pain treatment in the US leading to the opioid overdose crisis uncovered in 2017/18. In addition, over the counter drugs like ibuprofen can provoke severe side effects such as gastrointestinal bleedings. Hence, a better understanding of pain-inducing mechanisms as well as the development of novel targets for acute and chronic inflammatory pain treatment is essential.

Pain can be initiated and maintained by a subpopulation of primary sensory neurons, the nociceptors, which have their cell bodies in dorsal root ganglia (5, 6). According to the *International Association for the Study of Pain* (IASP), nociceptors are defined as: ‘A high-threshold sensory receptor of the peripheral somatosensory nervous system that is capable of transducing and encoding noxious stimuli’ (7). Peripheral branches of these pseudounipolar dorsal root ganglion neurons sense physical and chemical stimuli. After passing the dorsal root ganglion, central branches transmit the sensory information to the spinal cord. Nociceptive dorsal root ganglion neurons mostly have small-diameter cell bodies and are primarily responsible for slow pain sensation evoked by noxious stimuli (6). Chronic pain often results from temporary to permanent changes in the signaling cascades responsible for nociception. This leads to prolonged and enhanced transmission of nociceptive signals from the periphery to the central nervous system. For instance, the local inflammatory environment can sensitize nociceptors, increase the spontaneous action potential firing rate, and facilitate the responsiveness to endogenous or exogenous, proalgesic irritants (8).

**Figure 1:**
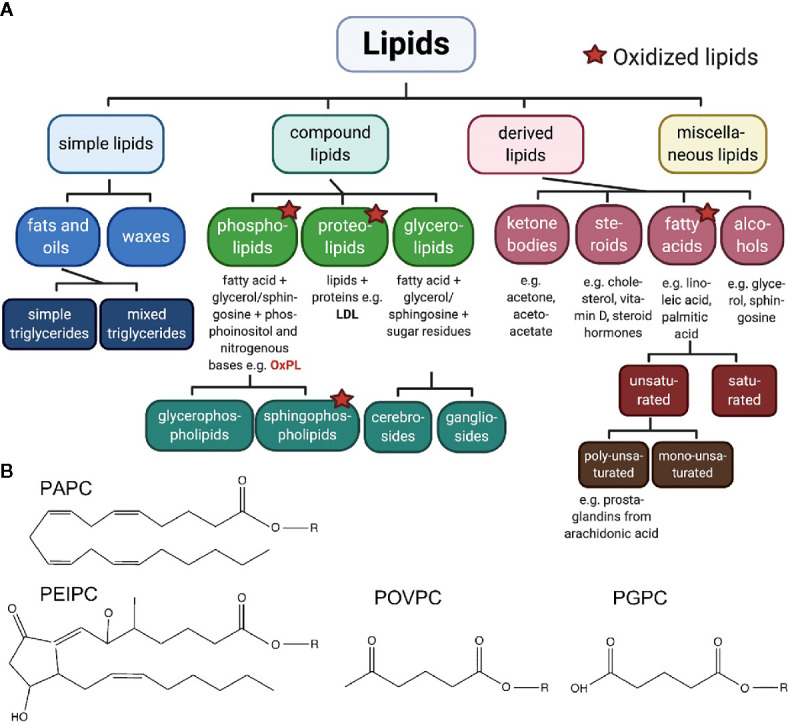
**(A)** Classification of lipids. The large group of lipids can be divided in four groups with respective subgroups. Oxidized phospholipids, pain-inducing, natural metabolites, are discussed in this review. Created with biorender.com^©^. **(B)** Pain-related oxidized phospholipids. The unoxidized PAPC consists of a 1‐palmitoyl‐sn‐glycero‐3‐phosphocholine backbone (R) and a linear, arachidonic tail of 20 carbon atoms including four double bonds. Oxidation of this phospholipid generates fragments such as POVPC and PGPC. In both molecules, the arachidonic tail is shortened to C5. Both molecules carry an aldehyde group or a carboxyl group, respectively. In addition, PEIPC is generated from PAPC by formation of a bond between C8 and C12, within the arachidonic tail, by reduction of two double bounds and additional oxygenation as well as radical formation.

**Figure 2:**
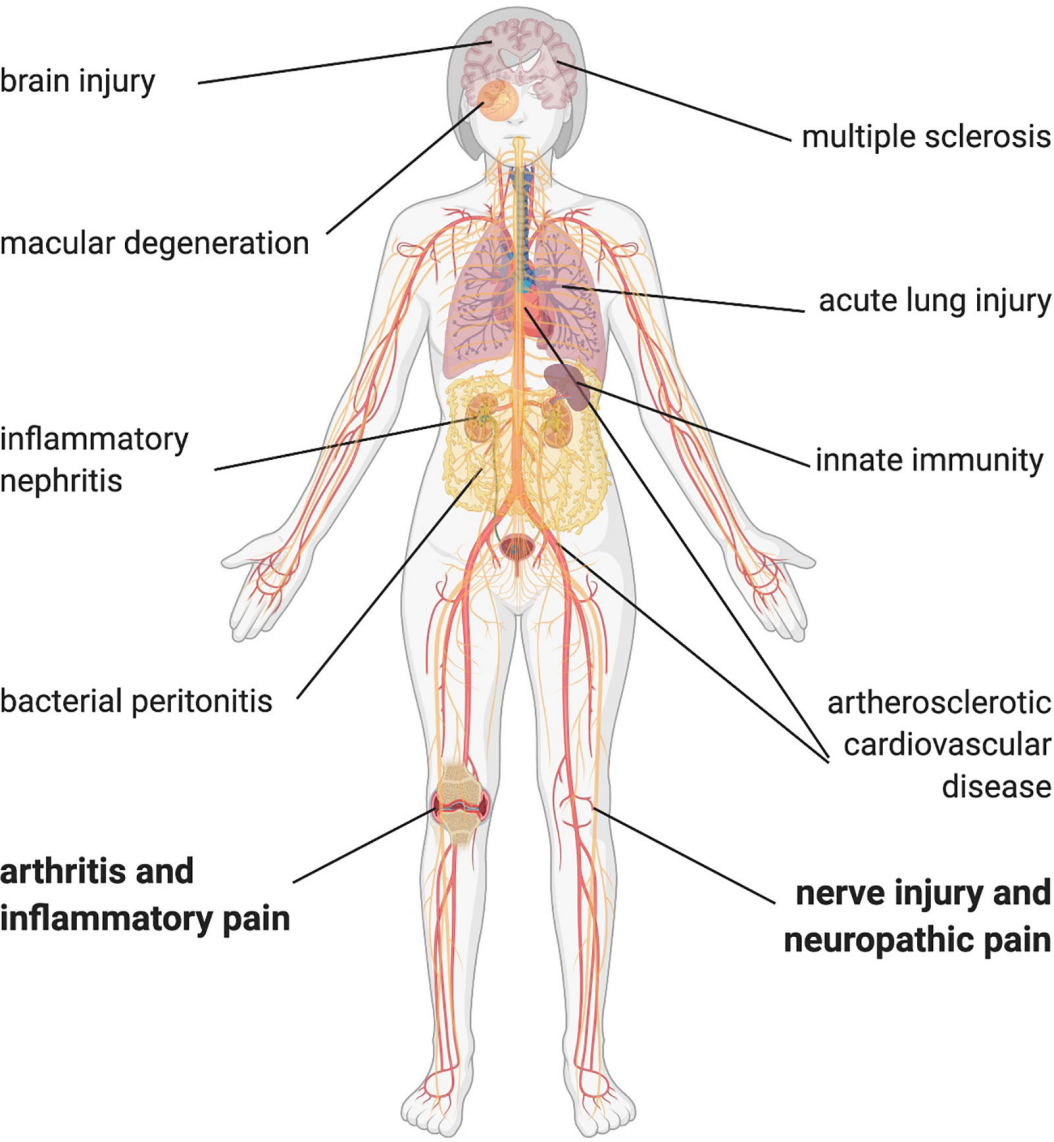
OxPL contributing to disease pathophysiology. OxPL can be found in several tissue affected by inflammatory diseases throughout the body. Most of the evidence comes from preclinical models, but especially in atherosclerotic cardiovascular disease and multiple sclerosis, there is evidence of OxPL in human tissue. Created with biorender.com^©^.

Recent research on lipids points toward its new role in pain signaling. Molecular components that act as pro- and analgesic factors, are found within the epilipidome. When looking at lipids in a hierarchical order (**Figure 1A**), compound lipids such as the ubiquitous phospholipids or glycerophospholipids, both critically important for integrity and function of all cellular membranes (9), are identified as upstream pain-inducing metabolites (10, 11). Phospholipids carry unsaturated fatty acids making them accessible for oxidation, nitration, and subsequent oxidative degradation. Chemical, non-enzymatic production of oxidized phospholipids (OxPL) leads to diverse biologically active OxPL species (proalgesic metabolites are indicated in **Figure 1B**). Besides non-enzymatic oxidation of phospholipids, enzymatic activity, for instance by lipoxygenases, also regulates OxPL abundance (9, 12, 13). Experimental evidence, mostly in preclinical rodent models, has corroborated the view that OxPL contribute to many diseases, including diverse pain syndromes, thus, making them attractive for a broad range of therapeutic approaches (**Figure 2**).

This review focuses on the biology of oxidized phospholipids (specifically in pain syndromes) and summarizes recent data in preclinical rodent pain models that show how targeting the biological activity of OxPL can control pain or can even contribute to natural pain resolution.

## Oxidized Phospholipids Are Linked to Inflammatory Conditions and Pain

Research on inflammatory pain in the early years focused on stable biomolecules like prostaglandins and peptides/proteins such as cytokines which trigger the action potential firing of nociceptors (8). Recently, works by our group and others have identified OxPL as proalgesic compounds in preclinical pain models (10, 11, 14, 15). Mechanistically, the highly reactive, transient, endogenous irritants directly activate ion channels on nociceptive C-fiber neurons. This function is different to the sensitizing effects provoked by typical inflammatory mediators (10, 11). Ion channels, like transient receptor potential ankyrin 1 (TRPA1) or voltage-gated sodium channels like Na_V_1.9, are exciting pharmacological targets for pain relief. Inhibiting ion channel function can stop effectively the transmission of nociceptive signals toward the central nervous system, devoid of central nervous system side effects. Therapeutic strategies against OxPL-mediated pain aim to reduce their direct excitatory function on nociceptors.

Acute and chronic inflammation can cause a variety of pain states. By affecting many different organs and the contribution to chronification of pain, inflammation is hindering pain resolution. Immune cells continuously produce reactive oxygen species (ROS), a source of highly reactive hydroxyl radicals. The reactions of ROS with phospholipids in plasma membranes and in lipoproteins lead to a continuous and even self-perpetuating production of OxPL (16). For instance, in inflammatory and neuropathic conditions such as arthritis or sciatic nerve axotomy, levels of a variety of reactive lipids rise in the plasma and in dorsal root ganglion neurons (17–19). Furthermore, levels of reactive lipids correlate positively with nocifensive behavior in mice. In endometriosis oxidized LDL (OxLDL) levels increase and subsequently occurring fragments such as prostaglandins evoke pain. In addition, OxLDL correlate with symptom severity in patients with fibromyalgia (20, 21). However, oxidized LDL levels remain comparable in patients with and without polyneuropathy due to type 2 diabetes (22). Notably, OxPL levels also rise in many other diseases (13). These include classical inflammatory diseases like peritonitis, nephritis, lung injury and multiple sclerosis (**Figure 2**). Most of the evidence comes from preclinical and clinical studies in cardiovascular disease (12, 23, 24). Many other bioactive lipids, e.g., eicosanoids (prostaglandins, leukotrienes, resolvins; see **Figure 1**) also rise already during the early acute phase of inflammation. It is obvious that both OxPL and eicosanoids co-exist in acute pain. It might be that OxPL, when generated non-enzymatically, appear a little bit earlier than eicosanoids. As some eicosanoids such as prostaglandin E2 are pro-inflammatory and are able to sensitize nociceptors and OxPL-mediators (e.g., Na_V_1.9), it might be beneficial to target non-enzymatic OxPL production as well as cyclooxygenases as early as possible.

## OxPAPC, a Mixture of Pain-Inducing OxPL Metabolites

Oxidation of PAPC (1-palmitoyl-2-arachidonoyl-sn-glycero-3-phosphorylcholine) generates long-chain and fragmented oxidized PAPC (OxPAPC) products. These include POVPC (1-palmitoyl-2-(5’-oxo-valeroyl)-sn-glycero-3-phosphocholine), PGPC (1-palmitoyl-2-glutaryl-sn-glycero-3-phospho-choline) and PEIPC (1-palmitoyl-2-5,6-epoxyisoprostane E2-sn-glycero-3-phosphatidyl-choline) (**Figure 1B**) (13). Commercially available OxPAPC is a mixture of molecules that is produced by air oxidation of synthetic PAPC. This OxPAPC-formulation serves as model substance for investigating OxPL functions in pain (10, 11).

OxPL can also be produced by enzymatic reactions, e.g., by lipoxygenases, cyclooxygenases, cytochrome P450 and cytochrome *c*. Enzymatic OxPL synthesis leads to a more precise formation of individual, often truncated, oxidized lipids (25). An enzymatic modification of PAPC has also been reported (26). How enzymatic production of phospholipids contributes to pain is not known yet, but it can well be that especially secreted lipoxygenases promote local, continuous and long-lasting OxPL production. From a mechanistic point of view, enzymatic OxPL production might even contribute to pain sensation in case of bacterial infections, because microbes such as Pseudomonas aeruginosa, a critical human pathogen responsible for many healthcare-associated infections, secrete lipoxygenases to produce OxPL (27).

OxPAPC as well as its corresponding metabolites excite nociceptors and pain behaviour in different models for inflammatory pain (10, 11, 14, 15, 28, 29). Specific OxPAPC components such as PGPC and POVPC as well as the OxPL degradation product lysophosphatidylcholine (LPC) are increased in chloroform-based lipid extracts from inflamed tissue by MALDI-TOF (10). Highly reactive, long-chain OxPL, such as PEIPC or POVPC, degrade to transient products or rearrange to more stable metabolites. These degradation products also show biological activity and include molecules such as LPC, the oxidation end product PGPC, the fragmented product 4-hydroxynonenal (4-HNE), or prostaglandin 15d-PGJ2 (10, 30). The ladder is a remote metabolite of OxPAPC. In addition to oxidative processes, enzymatic reactions, e.g., by phospholipase A2, facilitate the metabolization of OxPL species. The fast metabolization of the compounds produces new physiologically relevant, bioactive signaling molecules. Important is that many of the OxPL metabolites generated under inflammatory conditions can induce pain by acting as acute excitants.

## OxPL-Mediated Signal Transduction

**Figure 3:**
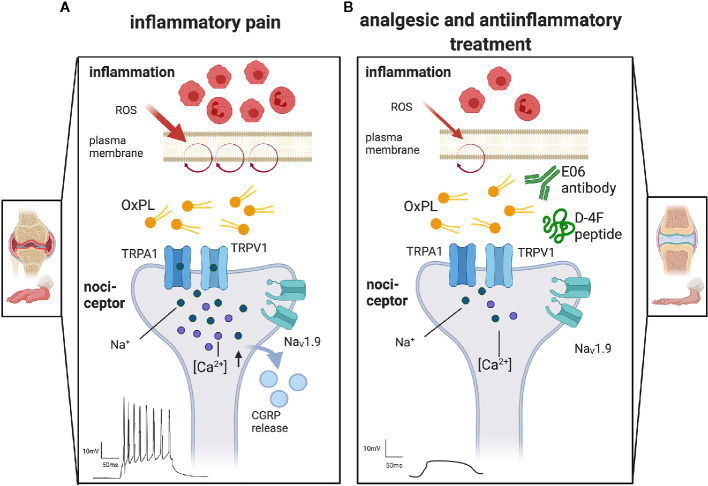
The proalgesic role of oxidized phospholipids and therapeutic options to target OxPL. Display of representative terminals of nociceptors, the first neuron in the pain pathway, found in peripheral tissue. **(A)** Inflammation attracts immune cells invading the tissue, e.g., in arthritis. Especially neutrophils and macrophages release reactive oxygen species (ROS). They oxidize lipids like those found in the plasma membrane (purple circle). The resulting oxidized phospholipids (OxPL) activate non-selective, excitatory ion channels like transient receptor potential ankyrin 1 (TRPA1) or transient receptor potential vanilloid 1 (TRPV1). Activation of TRP channels leads to a subsequent activation of voltage gated sodium channels (Na_V_) and the release of proalgesic molecules like calcitonin gene related peptides (CGRP). Action potentials are induced and propagated along the pain pathways, resulting in pain perception. **(B)** Treatment with monoclonal E06 antibodies or with the ApoA-I mimetic peptide D-4F scavenge OxPL and thereby reduce nociceptor firing, pain and inflammation. Created with biorender.com^©^.

The transient receptor potential channels TRPA1 and TRPV1 are excitatory ion channels mediating OxPL effects (**Figure 3**). They are polymodal, ionotropic receptors detecting noxious stimuli including chemical (e.g., capsaicin) or physical stimuli (e.g., heat) (5). Both cation channels are highly expressed in sensory neurons. When activated, the channels mediate pain behaviour and promote inflammation by triggering the increased release of neurotransmitters, inflammatory mediators and neuropeptides (8). Behavioural studies in knock-out mice and *in vitro* experiments on cultured small-diameter dorsal root ganglion neurons revealed that TRPA1 is the main target of OxPAPC and its components PGPC and POVPC. Much higher concentrations of the OxPL compounds are needed to stimulate TRPV1 responses and the induced activation of TRPC5, another TRP channel involved in pain mediation, is only short-lasting (10, 11, 31).

When applied rapidly and locally to cultured small-diameter neurons the prototypical OxPL compound PGPC induces calcium spikes – an effect mediated by TRPA1, but not TRPV1 (29). This is not easy to understand but points to a different mechanism of how OxPL activate TRP channels. For instance, TRPA1 activation by OxPL *via* an interaction with cysteines in the TRP N-terminus requires an electrophilic substance, similar to activation by the mustard oil compound AITC or 4-HNE or electrophilic OxPAPC compounds (28, 32, 33). Electrophilic compounds such as 4-HNE covalently bind *via* Michael addition and Schiff base formation to histidine, lysine, and cysteine residues, a key mechanism of TRPA1 activation. However, in an OxPAPC-hTRPA1 peptide binding study, no adduct formation and direct thiol oxidation were detected, suggesting that OxPAPC act as two-electron oxidants. However, Kelch-like ECH-associated protein 1 (Keap1), the oxidative stress sensor, is activated by the same electrophilic compounds as TRPA1 including OxPAPC (34).

Due to its chemical properties, the prototypical OxPL PGPC, a stable but non-electrophilic, acid oxidation end-product of OxPAPC, must use a different mechanism to activate TRPA1 (29). PGPC and other oxidized phospholipids rapidly integrate into the lipid bilayer of plasma membranes and thereby modulate functional properties of lipids and proteins (35). At least some of the OxPL compounds or even other members of the epilipidome probably induce rearrangements of the plasma membrane to mechanically activate TRPA1 and maybe even TRPV1 (36–38). When co-expressed, TRPA1 function can be increased *via* TRPV1-induced calcium influx. Though, an interaction of TRPA1 and TRPV1 might contribute to the rather complex OxPAPC action (see below).

Fast calcium imaging and patch-clamp recording revealed additional relevant ion channels that are involved in the signal transduction of OxPL stimuli (29). The unique low-threshold sodium channel Na_V_1.9 is of outstanding importance. In nociceptors, Na_V_1.9 is switched on under inflammatory conditions, by coincident signaling pathways (29, 39). When active, Nav1.9 potentiates acute OxPL responses and increases the excitability of neurons (29). This even induces calcium spikes in nociceptors that can be blocked with Ω -conotoxin, a high-affinity blocker of N-type voltage-gated calcium channels (29, 40). This is remarkable as N-type calcium channels mediate rather strong calcium influx signals. Activity-dependent calcium influx is often upstream of cellular plasticity effects such as activity-dependent axon growth (40) and is able to stimulate long-term changes in gene expression. It will be interesting to find out whether this calcium influx is involved in gene expression changes or axon sprouting upstream of nociceptor sensitization. Also, downstream, the stimulation of TRP channels with OxPAPC is sufficient to release calcitonin gene-related peptide (CGRP), a potent vasodilator and mediator of peripheral nociceptor sensitization. This indicates that OxPL are not only excitants, but also contribute indirectly to neurogenic inflammation (10, 15).

In summary, OxPL are nociceptor excitants and trigger signaling cascades that are able to induce long-term changes in nociceptors. Interrupting acute OxPL signaling might support pain resolution in transient and chronic inflammatory and possibly also neuropathic pain.

OxPL act locally and acutely on nociceptors. Biochemically, proalgesic OxPL are produced in all cellular membranes. It is likely that OxPL act autocrine as well paracrine. But, OxPL are also highly enriched in apolipoproteins. OxPL-rich apolipoproteins can also be found in the cerebrospinal fluid in the brain, spinal cord and dorsal root ganglion. Therefore, it is theoretically possible that OxPL contribute systemically to certain pain syndromes, maybe *via* a dysbalanced OxPL/apolipoprotein/cholesterol axis.

## Additional Oxidized Lipids Involved in Pain Perception

Besides the long-chain OxPL, other lipid metabolites being generated in inflammation evoke nocifensive behaviour in rodents. For instance, fragmented products of OxPAPC such as eicosanoids and prostaglandins are downstream of phospholipase A2-mediated conversion of arachidonic acid. Oxidized metabolites of the arachidonic and linoleic acids increase upon systemic stimulation mice with nerve growth factor, a most important neurotrophic factor involved in pain signaling (41, 42). In sterile inflammation, evoked by physical burn as well as UVB-mediated sunburn, derivates of oxidized linoleic acid increase (43, 44). Lysophosphatidic acid 18:1 and lysophosphatidyl choline 16:0 and 18:1, additional metabolites deriving from phosphatidylcholine precursors, mediate neuropathic pain (45–48). In a mouse model of chronic inflammation, oxidized linoleic acid-derived mediators increase in the central nervous system (49). The complex mode of actions in algesia are highlighted by modulation of small G-proteins such as GPR132 in addition to direct ion channel activation (18). These findings indicate that a variety of alterations of the lipid signaling cascade have deciphered promising approaches for future pain treatment.

## Targeting Oxidized Phospholipids to Treat Pain Syndromes

Advances in lipidomics give the opportunity to identify lipids that may serve as biomarkers or treatment targets. Based on the idea that proalgesic OxPL are preferentially produced locally and non-enzymatically, OxPL neutralization was thought to be a feasible strategy to target the proalgesic function of OxPL, at least in rodent pain models (10, 14, 15). Targeting enzymatic OxPL production with small molecule inhibitors, for instance oxazolone-derived compounds for lipoxygenase inhibition, is antinociceptive (50). It could well be that co-treatment with OxPL scavengers together with small molecule lipoxygenase blockers can synergistically even enhance the efficiency of pain control.

## Therapeutic Effects of Monoclonal E06 Antibodies for Pain Treatment

Oxidation of the phosphatidylcholine headgroup creates “neo”-self-antigens that are recognized by the acquired immune system. Distinct chemical structures and the conformation of lipids are indispensable for the formation of pattern recognising molecules such as antibodies. Anti-OxPL antibodies were originally discovered in preclinical models for atherosclerosis and pneumonia where they substantially increased under inflammatory conditions and (51, 52). One class of antibodies, the monoclonal E0 antibodies, were isolated from apoE-deficient mice, their eponym. Due to its high affinity to the oxidized head group of phosphatidylcholines, the monoclonal antibody (mAb) E06, also known as T15, got striking attention (53–55). Due to its unique properties, mAb E06 became an often-used tool to investigate OxPL abundance in various inflammatory diseases like acid-induced lung injury, arteriosclerosis, bacterial peritonitis, multiple sclerosis, inflammatory nephritis, and age-related macular degeneration (52, 56–60). Furthermore, the antibody was employed in immunohistochemistry or ELISA for OxPL binding studies. Notably, the mAb E06 also has therapeutic potential: passive immunization with E06 protects against atherosclerotic plaque development and inflammatory arthritis (61, 62).

In a model of inflammatory pain using complete Freund’s adjuvant (CFA), an E06-based competitive binding assay allowed for quantification of OxPAPC species in freshly isolated tissue. These experiments revealed rather high amounts of mAb E06 reactive OxPL in inflamed tissue (15, 28). These observations raised the idea that the pro-inflammatory OxPL function could also be blocked by antigen neutralization with the mAb E06. Indeed, locally applied E06 allowed to control pain behaviour in preclinical pain models (15, 28). Intraplantar application of E06 even ameliorated hypersensitivity in CFA-induced hind paw inflammation and collagen-induced arthritis (10). Furthermore, E06, but not its isotype control IgM, reduced the levels of low mass metabolization products of OxPAPC, like lysophosphatidylcholine (LPC 16:0).

In order to nail down specific mediators interacting with E06, TRPA1 and TRPV1 ligands were tested. *In vitro*, E06 prevented 4-HNE, OxPAPC, AITC, but not capsaicin-induced calcium influx in HEK-293 cells expressing recombinant TRPA1 and TRPV1. Furthermore, E06 could also inhibit corresponding TRPA1 and TRPV1 responses in dorsal root ganglion neurons. Mechanical hypersensitivity induced by intraplantar OxPAPC, or irritants like AITC or 4-HNE, was also prevented by local E06 application (15, 28). This was not due to binding of 4-HNE itself, but rather the formation of E06-reactive OxPLs after local irritant injection (15, 54).

Recently, in a series of very elegant experiments, the single-chain, non-immunogenic variable fragment of E06 (E06-scFv) has been expressed in transgenic mice. These experiments showed a striking potential of E06 to ameliorate atherosclerosis, non-alcoholic steatohepatitis, and high fat-induced bone loss (63–65). In summary, E06 is an exciting tool to scavenge ROS-induced downstream mediators and is a promising therapeutic for inflammatory diseases and inflammatory pain.

## D-4F, an ApoA-I Mimetic Peptide Shows Analgesic Potential

OxPL are typically transported by apolipoproteins, which can provide anti-atherogenic and anti-inflammatory properties. In order to mimic this function, apolipoprotein A-I (ApoA-I)-mimetic peptides have been developed. Especially the peptide D-4F, a small peptide of 2.3 kDa, shows considerable anti-inflammatory features *in vitro* and in animal models (66, 67). In addition, multiple doses of oral D-4F lower the HDL inflammatory index in high-risk coronary heart disease patients (68). D-4F has high affinity to OxPL – actually several magnitudes higher than ApoA-I. Apo-mimetic peptides, however, are not specific for OxPAPC: the binding capacity of D-4F to PGCP, POVPC, PEIPC and 1-(palmitoyl)-2-(5-keto-6-octene-dioyl) phosphatidylcholine (KOdiaA-PC) is substantially higher than for PAPC or other non-oxidized lipids, cholesterol, or oxidized lipids like 5(S)-hydroperoxy-5Z,8Z,10E,14Z-eicosatetraenoic acid (HPETE). Hence, the peptide D-4F is a well-suited tool to scavenge and neutralize free OxPL (67). As OxPL are involved in many diseases (**Figure 2**) and due to the fact that D-4F mimic a natural and common mechanism, it was suggested that these peptides could have a rather broad therapeutic potential. However, albeit the basic concept is rather old, mimicking the beneficial role of HDL/Apo-A1 or OxPL scavenging with mimetic peptides has not reached the clinic yet.

D-4F blocks H_2_O_2_, 4-HNE, and OxPAPC, but not capsaicin- or AITC-induced calcium influx in TRPA1- or TRPV1-expressing HEK293 cells or dorsal root ganglion neurons (10, 14). Apart from OxPL scavenging, other mechanisms of function are discussed for D-4F. It may be that 4-HNE, a lipid peroxidation downstream product, is the functional target of D-4F. 4-HNE could possibly bind to the lysine residues in D-4F and consecutively being inactivated (14). Indeed, in a murine asthma model, 4-HNE production was decreased after D-4F treatment. Alternatively, D-4F stimulates cholesterol efflux from cellular membranes, a mechanism altering lipid rafts and thereby nociceptors excitability (69, 70). Similar mechanisms have been observed in experiments with the ApoA-I binding protein which reduces lipid raft abundance *via* increased removal of excess cholesterol, thereby preventing allodynia in different preclinical models (71).

When D-4F is applied systemically, it inhibits capsaicin-, OxPAPC- and 4-HNE-evoked hypersensitivity in rodents (14, 28). In these experiments, D-4F was more effective against mechanical than thermal hypersensitivity. In preclinical models of inflammation like collagen-induced arthritis and CFA-induced hind paw inflammation, systemic D-4F reverses mechanical and thermal hypersensitivity as paw edema (14, 28).

In summary, D-4F is an analgesic substance ameliorating inflammatory pain. Whether it is beneficial in other painful diseases like traumatic nerve injury or chemotherapy-induced neuropathy needs further evaluation. Likewise, its exact mechanisms of function other than OxPAPC scavenging, e.g., interference with cholesterol metabolism like ABC transporters, and its potential in clinical trials have to be examined in the future.

## Conclusion

This review highlights oxidized phospholipids as novel targets for the treatment of acute and prolonged inflammatory pain. New technological developments allow an in-depth classification of oxidized lipids within the epilipidome and, thereby, the identification of innovative biomarkers and druggable targets. Detection of undiscovered compounds by oxidized lipidomics provide new insights into pain-generating mechanisms. Research shows that endogenous OxPL, generated under inflammatory conditions, activate well-studied pain pathways like the TRPA1 signaling cascade. Deciphering their modes of action improve the development of pharmacological tools like the monoclonal antibody E06 and the ApoA-1 mimetic peptide D-4F as promising new therapeutic options. E06 antibodies as well as ApoA1 are physiologically present in our body. This may point to the body’s own potential to handle pain and give a better understanding of the fundamental mechanisms of OxPL physiology. Insights might open new gates for pain management by strengthening natural mechanisms of pain resolution. Proven biological compatibility of D-4F in humans opens the possibility of local or systemic application of D-4F for patients with inflammatory pain, in the near future.

As our understanding of anti-OxPL therapy in pain has only been tested in preclinical rodent models in a few studies, mostly by a few research groups (10, 11, 14, 15, 29), a broad evaluation of the OxPL biology and anti-OxPL therapy by the science community is essentially needed.

## Author Contributions

BO drafted, wrote, and revised the manuscript. AB revised the manuscript. RB conceptualized, wrote, and revised the manuscript. HR created the graphics, wrote, and revised the review. All authors contributed to the article and approved the submitted version.

## Funding

Our studies on OxPL were funded by the Interdisciplinary Centre for Clinical Research (IZKF), University Hospital Würzburg, grants N-261 to BO, RB, and HR; Z2/CSP-2 to BO; and N-D-368 to RB and HR as well as by the Deutsche Forschungsgemeinschaft (DFG, German Research Foundation): project ID: 426503586, KFO5001 ResolvePAIN.

## Conflict of Interest

The authors declare that the research was conducted in the absence of any commercial or financial relationships that could be construed as a potential conflict of interest.

## Abbreviations

4-HNE, 4-hydroxynonenal; 13-HODE, 13-hydroxy-10E,12Z-octadecadienoic acid; 9-HODE, 9-hydroxy-10E-,12Z-octadecadienoic acid; ApoA-I, Apolipoprotein A-I; D-4F, D-amino-4-phenylalanine mimetic peptide of Apolipoprotein A-I; E06, E06 monoclonal antibody; H_2_O_2_, Hydrogen peroxide; HDL, High density lipoprotein; LDL, Low density lipoprotein; LPC, Lysophosphatidycholin; OxLDL, Oxidized low density lipoprotein; OxPAPC, Oxidized 1-palmitoyl-2-arachidonyl-sn- glycero-3-phosphorylcholine; OxPL, Oxidized phospholipids; PEIPC, 1-palmitoyl-2-(5,6)-epoxyisoprostane E2-sn-glycero-3-phosphocholine; PGPC, 1-palmitoyl-2-glutaryl phosphatidylcholine; POVPC, 1-palmitoyl-2-(5-oxovaleroyl)-sn-glycero-3-phosphocholine; ROS, Reactive oxygen species; TLR-4, Toll-like receptor 4; TRPA1, Transient receptor potential ankyrin type 1; TRPC5, Transient receptor potential canonical type 5; TRPV1, Transient receptor potential vanilloid type 1.

## References

[1] Merskey H Bogduk N IASP Terminology. In: IASP Task Force on Taxonomy, 2 Seattle: IASP press (1994). p. 209–14.

[2] Kuehn B Chronic Pain Prevalence. JAMA (2018) 320(16):1632–2. 10.1001/jama.2018.16009 30357307

[3] Macfarlane G The epidemiology of chronic pain. Pain (2016) 157(10):2158–9. 10.1097/j.pain.0000000000000676 27643833

[4] Treede R-D Rief W Barke A Aziz Q Bennett MI Benoliel R Chronic pain as a symptom or a disease: the IASP Classification of Chronic Pain for the International Classification of Diseases (ICD-11). Pain (2019) 160(1):19–27. 10.1097/j.pain.0000000000001384 30586067

[5] Basbaum AI Bautista DM Scherrer G Julius D Cellular and Molecular Mechanisms of Pain. Cell (2009) 139(2):267–84. 10.1016/j.cell.2009.09.028 PMC2852643 19837031

[6] Dubin AE Patapoutian A Nociceptors: the sensors of the pain pathway. J Clin Invest (2010) 120(11):3760–72. 10.1172/JCI42843 PMC2964977 21041958

[7] Cohen M Quintner J van Rysewyk S Reconsidering the International Association for the Study of Pain definition of pain. Pain Rep (2018) 3(2):e634. 10.1097/PR9.0000000000000634 29756084 PMC5902253

[8] Ji R-R Xu Z-Z Gao Y-J Emerging targets in neuroinflammation-driven chronic pain. Nat Rev Drug Discovery (2014) 13(7):533–48. 10.1038/nrd4334 PMC4228377 24948120

[9] Bochkov VN Oskolkova OV Birukov KG Levonen A-L Binder CJ Stöckl J Generation and biological activities of oxidized phospholipids. Antioxid Redox Signal (2010) 12(8):1009–59. 10.1089/ars.2009.2597 PMC3121779 19686040

[10] Oehler B Kistner K Martin C Schiller J Mayer R Mohammadi M Inflammatory pain control by blocking oxidized phospholipid-mediated TRP channel activation. Sci Rep (2017) 14 7(1):5447. 10.1038/s41598-017-05348-3 PMC5511297 28710476

[11] Liu B Tai Y Caceres AI Achanta S Balakrishna S Shao X Oxidized Phospholipid OxPAPC Activates TRPA1 and Contributes to Chronic Inflammatory Pain in Mice. PLoS One (2016) 11(11):e0165200. 10.1371/journal.pone.0165200 27812120 PMC5094666

[12] Nie J Yang J Wei Y Wei X The role of oxidized phospholipids in the development of disease. Mol Aspects Med (2020) 76:100909. 10.1016/j.mam.2020.100909 33023753

[13] Fruhwirth GO Loidl A Hermetter A Oxidized phospholipids: From molecular properties to disease. Biochim Biophys Acta BBA Mol Basis Dis (2007) 1772(7):718–36. 10.1016/j.bbadis.2007.04.009 17570293

[14] Oehler B Kloka J Mohammadi M Ben-Kraiem A Rittner HL. D-4F, an ApoA-I mimetic peptide ameliorating TRPA1-mediated nocifensive behaviour in a model of neurogenic inflammation. Mol Pain (2020) 16:1744806920903848. 10.1177/1744806920903848 31996074 PMC6993174

[15] Mohammadi M Oehler B Kloka J Martin C Brack A Blum R Antinociception by the anti-oxidized phospholipid antibody E06. Br J Pharmacol (2018) 175(14):2940–55. 10.1111/bph.14340 PMC6016623 29679953

[16] Freigang S The regulation of inflammation by oxidized phospholipids. Eur J Immunol (2016) 46(8):1818–25. 10.1002/eji.201545676 27312261

[17] Osthues T Sisignano M Oxidized Lipids in Persistent Pain States. Front Pharmacol (2019) 10:1147. 10.3389/fphar.2019.01147 31680947 PMC6803483

[18] Hohmann SW Angioni C Tunaru S Lee S Woolf CJ Offermanns S The G2A receptor (GPR132) contributes to oxaliplatin-induced mechanical pain hypersensitivity. Sci Rep (2017) 7(1):446. 10.1038/s41598-017-00591-0 28348394 PMC5428564

[19] Sarban S Kocyigit A Yazar M Isikan UE Plasma total antioxidant capacity, lipid peroxidation, and erythrocyte antioxidant enzyme activities in patients with rheumatoid arthritis and osteoarthritis. Clin Biochem (2005) 38(11):981–6. 10.1016/j.clinbiochem.2005.08.003 16150434

[20] Kutu FC Özdolap Ş Sarikaya S Pro-inflammatory Cytokines and Oxidized Low-Density-Lipoprotein in Patients With Fibromyalgia. Arch Rheumatol (2019) 34(2):123–9. 10.5606/ArchRheumatol.2019.6733 PMC6719580 31497758

[21] Ray K Fahrmann J Mitchell B Paul D King H Crain C Oxidation Sensitive Nociception Involved in Endometriosis Associated Pain. Pain (2015) 156(3):528–39. 10.1097/01.j.pain.0000460321.72396.88 PMC4329051 25599233

[22] Rosales-Hernandez A Cheung A Podgorny P Chan C Toth C Absence of clinical relationship between oxidized low density lipoproteins and diabetic peripheral neuropathy: a case control study. Lipids Health Dis (2014) 13(1):32. 10.1186/1476-511X-13-32 24520839 PMC3933384

[23] Boffa MB Koschinsky ML Oxidized phospholipids as a unifying theory for lipoprotein(a) and cardiovascular disease. Nat Rev Cardiol (2019) 16(5):305–18. 10.1038/s41569-018-0153-2 30675027

[24] Bartolini Gritti B Binder CJ Oxidation-specific epitopes are major targets of innate immunity in atherothrombosis. Hamostaseologie (2016) 36(2):89–96. 10.5482/HAMO-14-11-0069 25682990

[25] Davies SS Guo L Lipid peroxidation generates biologically active phospholipids including oxidatively N-modified phospholipids. Chem Phys Lipids (2014) 181:1–33. 10.1016/j.chemphyslip.2014.03.002 24704586 PMC4075969

[26] Bochkov V Gesslbauer B Mauerhofer C Philippova M Erne P Oskolkova OV Pleiotropic effects of oxidized phospholipids. Free Radic Biol Med (2017) 111:6–24. 10.1016/j.freeradbiomed.2016.12.034 28027924

[27] Aldrovandi M Banthiya S Meckelmann S Zhou Y Heydeck D O’Donnell VB Specific oxygenation of plasma membrane phospholipids by Pseudomonas aeruginosa lipoxygenase induces structural and functional alterations in mammalian cells. Biochim Biophys Acta BBA - Mol Cell Biol Lipids (2018) 1863(2):152–64. 10.1016/j.bbalip.2017.11.005 PMC5764228 29146531

[28] Oehler B Mohammadi M Perpina Viciano C Hackel D Hoffmann C Brack A Peripheral Interaction of Resolvin D1 and E1 with Opioid Receptor Antagonists for Antinociception in Inflammatory Pain in Rats. Front Mol Neurosci (2017) 10:242. 10.3389/fnmol.2017.00242 28824373 PMC5541027

[29] Martin C Stoffer C Mohammadi M Hugo J Leipold E Oehler B NaV1.9 Potentiates Oxidized Phospholipid-Induced TRP Responses Only under Inflammatory Conditions. Front Mol Neurosci (2018) 11:7. 10.3389/fnmol.2018.00007 29410612 PMC5787077

[30] Rittner HL Hackel D Voigt P Mousa S Stolz A Labuz D Mycobacteria Attenuate Nociceptive Responses by Formyl Peptide Receptor Triggered Opioid Peptide Release from Neutrophils. Bishai W, editor. PLoS Pathog (2009) 5(4):e1000362. 10.1371/journal.ppat.1000362 19343210 PMC2657213

[31] AL-Shawaf E Naylor J Taylor H Riches K Milligan CJ O’Regan D Short-Term Stimulation of Calcium-Permeable Transient Receptor Potential Canonical 5–Containing Channels by Oxidized Phospholipids. Arterioscler Thromb Vasc Biol (2010) 30(7):1453–9. 10.1161/ATVBAHA.110.205666 PMC2887788 20378846

[32] Hinman A Chuang H-h Bautista DM Julius D TRP channel activation by reversible covalent modification. Proc Natl Acad Sci (2006) 103(51):19564–8. 10.1073/pnas.0609598103 PMC1748265 17164327

[33] Macpherson LJ Dubin AE Evans MJ Marr F Schultz PG Cravatt BF Noxious compounds activate TRPA1 ion channels through covalent modification of cysteines. Nature 2007 (7127) 445:541–5. 10.1038/nature05544 17237762

[34] Ooi BK Goh BH Yap WH Oxidative Stress in Cardiovascular Diseases: Involvement of Nrf2 Antioxidant Redox Signaling in Macrophage Foam Cells Formation. Int J Mol Sci (2017) 18(11):2336. 10.3390/ijms18112336 PMC5713305 29113088

[35] Stemmer U Hermetter A Protein modification by aldehydophospholipids and its functional consequences. Biochim Biophys Acta BBA Biomembr (2012) 1818(10):2436–45. 10.1016/j.bbamem.2012.03.006 PMC3790970 22450235

[36] Kwan KY Allchorne AJ Vollrath MA Christensen AP Zhang D-S Woolf CJ TRPA1 Contributes to Cold, Mechanical, and Chemical Nociception but Is Not Essential for Hair-Cell Transduction. Neuron (2006) 50(2):277–89. 10.1016/j.neuron.2006.03.042 16630838

[37] Jansson ET Trkulja CL Ahemaiti A Millingen M Jeffries GD Jardemark K Effect of Cholesterol Depletion on the Pore Dilation of TRPV1. Mol Pain (2013) 9:1744-8069-9–1. 10.1186/1744-8069-9-1 PMC3560271 23279936

[38] Saghy E Szoke E Payrits M Helyes Z Borzsei R Erostyak J Evidence for the role of lipid rafts and sphingomyelin in Ca-gating of Transient Receptor Potential channels in trigeminal sensory neurons and peripheral nerve terminals. Pharmacol Res (2015) 100:101–16. 10.1016/j.phrs.2015.07.028 26238178

[39] Maingret F Coste B Padilla F Clerc N Crest M Korogod SM Inflammatory Mediators Increase Nav1.9 Current and Excitability in Nociceptors through a Coincident Detection Mechanism. J Gen Physiol (2008) 131(3):211–25. 10.1085/jgp.200709935 PMC2248717 18270172

[40] Subramanian N Wetzel A Dombert B Yadav P Havlicek S Jablonka S Role of Nav1.9 in activity-dependent axon growth in motoneurons. Hum Mol Genet (2012) 21(16):3655–67. 10.1093/hmg/dds195 22641814

[41] Denk F Bennett DL McMahon SB Nerve Growth Factor and Pain Mechanisms. Annu Rev Neurosci (2017) 40(1):307–25. 10.1146/annurev-neuro-072116-031121 28441116

[42] Eskander MA Ruparel S Green DP Chen PB Por ED Jeske NA Persistent Nociception Triggered by Nerve Growth Factor (NGF) Is Mediated by TRPV1 and Oxidative Mechanisms. J Neurosci (2015) 35(22):8593–603. 10.1523/JNEUROSCI.3993-14.2015 PMC4452557 26041925

[43] Green D Ruparel S Gao X Ruparel N Patil M Akopian A Central activation of TRPV1 and TRPA1 by novel endogenous agonists contributes to mechanical allodynia and thermal hyperalgesia after burn injury. Mol Pain (2016) 12:1744806916661725. 10.1177/1744806916661725 27411353 PMC4955965

[44] Sisignano M Angioni C Ferreiros N Schuh CD Suo J Schreiber Y Synthesis of Lipid Mediators during UVB-Induced Inflammatory Hyperalgesia in Rats and Mice. PLoS One (2013) 8(12):e81228. 10.1371/journal.pone.0081228 24349046 PMC3857181

[45] Kuwajima K Sumitani M Kurano M Kano K Nishikawa M Uranbileg B Lysophosphatidic acid is associated with neuropathic pain intensity in humans: An exploratory study. Ikeda K, editor. PLoS One (2018) 13(11):e0207310. 10.1371/journal.pone.0207310 30408112 PMC6224112

[46] Ueda H LPA receptor signaling as a therapeutic target for radical treatment of neuropathic pain and fibromyalgia. Pain Manag (2020) 10(1):43–53. 10.2217/pmt-2019-0036 31852400

[47] Rimola V Hahnefeld L Zhao J Jiang C Angioni C Schreiber Y Lysophospholipids contribute to oxaliplatin-induced acute peripheral pain. J Neurosci (2020) 40(49):9519–32. 10.1523/JNEUROSCI.1223-20.2020 PMC7724144 33158961

[48] Ma L Nagai J Chun J Ueda H An LPA species (18:1 LPA) plays key roles in the self-amplification of spinal LPA production in the peripheral neuropathic pain model. Mol Pain (2013) 9(1):29. 10.1186/1744-8069-9-29 23773289 PMC3691926

[49] Jensen JR Pitcher MH Yuan ZX Ramsden CE Domenichiello AF Concentrations of oxidized linoleic acid derived lipid mediators in the amygdala and periaqueductal grey are reduced in a mouse model of chronic inflammatory pain. Prostaglandins Leukot Essent Fatty Acids (2018) 135:128–36. 10.1016/j.plefa.2018.07.015 PMC6269101 30103924

[50] Mavridis E Bermperoglou E Pontiki E Hadjipavlou-Litina D 5-(4H)-Oxazolones and Their Benzamides as Potential Bioactive Small Molecules. Mol Basel Switz (2020) 25(14):3173. 10.3390/molecules25143173 PMC7397336 32664550

[51] Briles DE Forman C Hudak S Claflin JL Anti-phosphorylcholine antibodies of the T15 idiotype are optimally protective against Streptococcus pneumoniae. J Exp Med (1982) 156(4):1177–85. 10.1084/jem.156.4.1177 PMC2186814 7153709

[52] Shaw PX Hörkkö S Chang M-K Curtiss LK Palinski W Silverman GJ Natural antibodies with the T15 idiotype may act in atherosclerosis, apoptotic clearance, and protective immunity. J Clin Invest (2000) 105(12):1731–40. 10.1172/JCI8472 PMC378505 10862788

[53] Friedman P Hörkkö S Steinberg D Witztum JL Dennis EA Correlation of Antiphospholipid Antibody Recognition with the Structure of Synthetic Oxidized Phospholipids: importance of schiff base formation and aldol condensation. J Biol Chem (2002) 277(9):7010–20. 10.1074/jbc.M108860200 11744722

[54] Palinski W Hörkkö S Miller E Steinbrecher UP Powell HC Curtiss LK Cloning of monoclonal autoantibodies to epitopes of oxidized lipoproteins from apolipoprotein E-deficient mice. Demonstration of epitopes of oxidized low density lipoprotein in human plasma. J Clin Invest (1996) 98(3):800–14. 10.1172/JCI118853 PMC507491 8698873

[55] Thimmulappa RK Gang X Kim J-H Sussan TE Witztum JL Biswal S Oxidized phospholipids impair pulmonary antibacterial defenses: Evidence in mice exposed to cigarette smoke. Biochem Biophys Res Commun (2012) 426(2):253–9. 10.1016/j.bbrc.2012.08.076 PMC3495329 22935414

[56] Buga GM Frank JS Mottino GA Hendizadeh M Hakhamian A Tillisch JH D-4F decreases brain arteriole inflammation and improves cognitive performance in LDL receptor-null mice on a Western diet. J Lipid Res (2006) 47(10):2148–60. 10.1194/jlr.M600214-JLR200 16835442

[57] Haider L Fischer MT Frischer JM Bauer J Hoftberger R Botond G Oxidative damage in multiple sclerosis lesions. Brain (2011) 134(7):1914–24. 10.1093/brain/awr128 PMC3122372 21653539

[58] Imai Y Kuba K Neely GG Yaghubian-Malhami R Perkmann T van Loo G Identification of Oxidative Stress and Toll-like Receptor 4 Signaling as a Key Pathway of Acute Lung Injury. Cell (2008) 133(2):235–49. 10.1016/j.cell.2008.02.043 PMC7112336 18423196

[59] Matt U Sharif O Martins R Furtner T Langeberg L Gawish R WAVE1 mediates suppression of phagocytosis by phospholipid-derived DAMPs. J Clin Invest (2013) 123(7):3014–24. 10.1172/JCI60681 PMC3696563 23934128

[60] Ravandi A Leibundgut G Hung M-Y Patel M Hutchins PM Murphy RC Release and Capture of Bioactive Oxidized Phospholipids and Oxidized Cholesteryl Esters During Percutaneous Coronary and Peripheral Arterial Interventions in Humans. J Am Coll Cardiol (2014) 63(19):1961–71. 10.1016/j.jacc.2014.01.055 PMC4629781 24613321

[61] Chen Y Khanna S Goodyear CS Park YB Raz E Thiel S Regulation of dendritic cells and macrophages by an anti-apoptotic cell natural antibody that suppresses TLR responses and inhibits inflammatory arthritis. J Immunol Baltim Md (1950) 2009 183(2):1346–59. 10.4049/jimmunol.0900948 PMC2713016 19564341

[62] Faria-Neto JR Chyu K-Y Li X Dimayuga PC Ferreira C Yano J Passive immunization with monoclonal IgM antibodies against phosphorylcholine reduces accelerated vein graft atherosclerosis in apolipoprotein E-null mice. Atherosclerosis (2006) 189(1):83–90. 10.1016/j.atherosclerosis.2005.11.033 16386745

[63] Ambrogini E Que X Wang S Yamaguchi F Weinstein RS Tsimikas S Oxidation-specific epitopes restrain bone formation. Nat Commun (2018) 9(1):2193. 10.1038/s41467-018-04047-5 29875355 PMC5990540

[64] Que X Hung M-Y Yeang C Gonen A Prohaska TA Sun X Oxidized phospholipids are proinflammatory and proatherogenic in hypercholesterolaemic mice. Nature 2018 (7709) 558:301–6. 10.1038/s41586-018-0198-8 PMC6033669 29875409

[65] Sun X Seidman JS Zhao P Troutman TD Spann NJ Que X Neutralization of Oxidized Phospholipids Ameliorates Non-alcoholic Steatohepatitis. Cell Metab (2020) 31(1):189–206.e8. 10.1016/j.cmet.2019.10.014 31761566 PMC7028360

[66] Navab M Anantharamaiah GM Reddy ST Hama S Hough G Grijalva VR Oral D-4F Causes Formation of Pre-β High-Density Lipoprotein and Improves High-Density Lipoprotein–Mediated Cholesterol Efflux and Reverse Cholesterol Transport From Macrophages in Apolipoprotein E–Null Mice. Circulation (2004) 109(25):3215–20. 10.1161/01.CIR.0000134275.90823.87 15197147

[67] Lenten BJV Wagner AC Jung C-L Ruchala P Waring AJ Lehrer RI Anti-inflammatory apoA-I-mimetic peptides bind oxidized lipids with much higher affinity than human apoA-I. J Lipid Res (2008) 49(11):2302–11. 10.1194/jlr.M800075-JLR200 PMC2563211 18621920

[68] Dunbar RL Movva R Bloedon LT Duffy D Norris RB Navab M Oral Apolipoprotein A-I Mimetic D-4F Lowers HDL-Inflammatory Index in High-Risk Patients: A First-in-Human Multiple-Dose, Randomized Controlled Trial. Clin Transl Sci (2017) 10(6):455–69. 10.1111/cts.12487 PMC5673907 28795506

[69] Amsalem M Poilbout C Ferracci G Delmas P Padilla F Membrane cholesterol depletion as a trigger of Nav1.9 channel-mediated inflammatory pain. EMBO J (2018) 37(8):e97349. 10.15252/embj.201797349 29459435 PMC5897772

[70] Xie Q Zhao S Li F D-4F, an apolipoprotein A-I mimetic peptide, promotes cholesterol efflux from macrophages via ATP-binding cassette transporter A1. Tohoku J Exp Med (2010) 220(3):223–8. 10.1620/tjem.220.223 20208418

[71] Woller SA Choi S-H An EJ Low H Schneider DA Ramachandran R Inhibition of Neuroinflammation by AIBP: Spinal Effects upon Facilitated Pain States. Cell Rep (2018) 23(9):2667–77. 10.1016/j.celrep.2018.04.110 PMC6239868 29847797

